# Free Plasma Amino Acid Concentrations in Horses Fed Different Dosing Regimens of Hydrolysed Collagen

**DOI:** 10.3390/ani15213195

**Published:** 2025-11-03

**Authors:** Lieuwke C. Kranenburg, Katharina S. Reinke, Jan van den Broek, Esther A. Zaal, Robin van den Boom, David A. van Doorn

**Affiliations:** 1 Department of Clinical Sciences, Faculty of Veterinary Medicine, Utrecht University, Yalelaan 112, 3584 CM Utrecht, The Netherlandsr.vandenboom@uu.nl (R.v.d.B.); 2 Department Biomolecular Health Sciences, Faculty of Veterinary Medicine, Utrecht University, Yalelaan 2, 3584 CM Utrecht, The Netherlands; 3 Department of Population Health Sciences, Farm Animal Health, Faculty of Veterinary Medicine, Utrecht University, Yalelaan 7, 3584 CL Utrecht, The Netherlands

**Keywords:** hydrolysed collagen, equine, oral availability, supplement

## Abstract

**Simple Summary:**

Dietary supplements containing hydrolysed collagen (HC) are used for the treatment of osteoarthritis and improvement of hoof horn growth in horses and more recently in the management of gastric ulcers in horses. The purpose of this study was to investigate the oral availability and determine the appropriate dose of a specific commercial equine dietary supplement containing HC, by measuring the free amino acid (AA) profiles in blood plasma. This study demonstrated the availability of the hydrolysed collagen of this specific dietary supplement and that 100 g/day resulted in higher plasma concentrations, which could be detected for at least 24 h, suggesting a potentially greater clinical relevance.

**Abstract:**

Hydrolysed collagen is used as a supplement for horses with osteoarthritis, hoof horn growth problems, and gastric ulcers. To determine the oral availability of a specific hydrolysed collagen supplement and the appropriate dose, six Warmblood mares were fed two different concentrations of the supplement: 100 g HC (C_H_), 50 g HC (C_L_), and a control of 0 g HC (C_N_) during one week in a randomised cross-over design. On day 7, 14 and 21, blood sampling for amino acid (AA) analysis was performed, just prior to feeding the supplement (t = 0) and every hour after feeding for 8 h (t = 1–8). Statistical analysis revealed differences in mean plasma AA concentrations between the CH and CN doses for alanine, arginine, glutamine, glycine, proline, serine and hydroxyproline. Similarly, statistical differences were observed between the CL and CN doses for arginine, glycine, proline and hydroxyproline. This study demonstrated the availability of amino acids from the supplemented hydrolysed collagen. Although clinical efficacy was not evaluated in this study, a dose of 100 g HC once daily resulted in higher plasma concentrations, which remained detectable for at least 24 h, suggesting greater clinical relevance.

## 1. Introduction

There is a growing interest in supplements for horses containing collagen or hydrolysed collagen (HC). HC supplements are used for the treatment of osteoarthritis in humans [1,2,3,4,5,6,7] and for reducing activity-related joint pain in athletes [8]. Hydrolysed collagen is also used in the treatment of osteoarthritis in dogs [9,10,11,12] and has been reported to have a positive effect on osteoarthritis in horses [13,14] and to support improvement of hoof horn growth [15,16]. Recently, the use of hydrolysed collagen in the management of Equine Gastric Ulcer Syndrome (EGUS) has also been evaluated [17,18], after earlier reports of the potential beneficial effect of collagen in gastric ulceration induced in rats [19,20,21].

It has been proposed that orally administered collagen substrates are digested in the equine stomach, and later in the small intestine, to di- and tripeptides and individual amino acids (AAs) [22]. These are absorbed via various transport systems across the enterocyte membranes into the bloodstream [23]. AAs detected in plasma confirm intestinal absorption and are therefore used as an indicator of oral availability. In humans hydroxyproline was present in plasma 1–2 h after oral administration of collagen hydrolysates, confirming systemic absorption [24].

Coenen et al. (2006) assessed the effect of one dose (60 g/horse/day) of gelatin in horses in training and showed that AAs from gelatin are quickly available [25]. However, it is unknown if plasma AAs respond in a dose-dependent manner. Therefore, the purpose of this study was to evaluate the oral availability of a specific commercial equine dietary supplement containing hydrolysed collagen by measuring the free amino acid profiles in blood plasma after a dose of 50 g/horse/day and 100 g/horse/day.

## 2. Materials and Methods

### 2.1. Study Design

Six university-owned horses were fed two different supplement regimens (C_L_ = 50 g/d hydrolysed collagen (C_H_ = 100 g/d hydrolysed collagen) and a control (C_N_ = 0 g/d hydrolysed collagen) in a randomised cross-over design. Horses received the supplement for 7 days per dose before sampling. On day 7, 14 and 21, blood sampling for amino acid (AA) analysis was performed just prior (t = 0) to and every hour after feeding of the supplement for 8 h (t = 1–8) (Table 1). This project was approved by the university’s ethical committee (AVD1080020185204-3-01).

### 2.2. Selection of Horses

The six horses were selected out of a herd of ten Warmblood mares (mean age 9.17 years ± SD 0.98, mean weight 590.67 kg ± SD 40.63, BCS 4.83 ± SD 0.75). The horses were selected based on a body condition score between 4 and 6 on a scale of 9 of the Henneke Body Condition scoring system [26], comparable age and weight, no known previous health problems and a clinical examination within normal limits. Subclinical disease was excluded by measuring the following blood parameters: haematocrit, white blood cell counts with differentiation, blood urea nitrogen, creatinine, gamma glutamyl transferase, alkaline phosphatase, creatine kinase, aspartate aminotransferase, lactate dehydrogenase and total protein. Before the start of the experiments all horses were subjected to a gastroscopy to rule out the presence of Equine Gastric Ulcer Syndrome (EGUS) [27]. Horses with an Equine Squamous Gastric Disease score of two or more or with Equine Glandular Gastric Disease were excluded. Six horses that did not show any abnormalities were included in the dose–response study.

During the study the clinical parameters body temperature, respiratory rate, heart rate, faecal consistency and behaviour were monitored daily. The mares’ bodyweight was measured once a week. The animals were housed individually at the University clinic, but they were able to see other horses. During the study period the exercise regime consisted of one hour in the horse walker per day.

### 2.3. Diet

All horses were fed to cover energy and protein requirements for low exercise intensity, as defined by the Dutch evaluation system for horses [28]. Prior to the study a forage analysis was performed (Eurofins Agro, Wageningen, The Netherlands). During the study the horses were fed 12 kg hay per day according to the recommendations of Harris et al. [29]. In addition, to meet the vitamin and mineral requirements of the animals, 0.6 kg of a balancer (Subli Mineralenbikkels^®^, Voermeesters B.V. Wageningen, The Netherlands) was added (Tables S1 and S2).

The horses were fed a control diet or received one of the two different doses of a specific hydrolysed collagen product (SONAC Hydro-P Premium^®^, Darling Ingredients, Son, The Netherlands) added to the same concentrate feed (Voermeesters Basis^®^, Voermeesters B.V., Wageningen, The Netherlands). The CH dosing regimens were obtained by providing control horses with pellet feed I (Voermeesters Basis^®^, Voermeesters B.V., Lienden, The Netherlands) without HC (C_N_) and a pelleted feed (II) that included HC (C_H_). The horses received 1000 g (C_N_), 1050 g (C_L_) or 1100 g CH, and this was fed to the horses at 08:00. The C_L_ regimen was realised by mixing the two pellets. The horses received the same supplement doses regardless of their bodyweight.

On day 7, 14 and 21 the horses did not receive forage or the vitamin and mineral supplement during the blood sampling. The horses only received the pellet feed with the dose regimen at 08:00 h adhering to the normal feeding schedule to eliminate any influence of other dietary components on the blood results. The last hay was fed at 20:00 h on the day prior to sampling.

### 2.4. Sampling

For blood sampling on day 7, 14 and 21, a 16 G polyurethane catheter (Extended Use MILACATH^®^—16 Ga × 13 cm (5.25 in), MILA International, Florence, SC, USA) was placed in the jugular vein of all horses. For easy access during sampling an injection stopper (IN-Stopper^®^ for intermittent injections through injection membrane yellow, Braun, Melsungen, Germany) was connected to the catheter. The catheter was fixed with a cross-stitch suture using ethylene 0 (Ethilon^TM^ 0 Ethicon, LLC, San Lorenzo, PR, USA) as suture material. Blood samples were collected at t0 = 07:45, t1 = 09:00, t2 = 10:00, t3 = 11:00, t4 = 12:00, t5 = 13:00, t6 = 14:00, t7 = 15:00 and t8 = 16:00. Per sampling 6 mL of blood was collected and divided over two lithium-heparinized tubes (Vacuette^®^ Tube 3.5 mL LH Lithium Heparin Separator, Greiner Bio-One, Alpen aan den Rijn, The Netherlands). To prevent blood clotting inside the catheters, these were flushed with 5 mL 0.9% sodium-chloride solution + heparin after each sampling. The samples were left for 60 min to allow blood to clot, preventing cellular components from being present in the final serum sample and ensuring accurate test results, before they were centrifuged (Rontana/S Centrifuge, Hettich, Tüttlingen, Germany) for 10 min at 2778 g. The plasma was harvested in 2 mL PCR tubes (PCR-PT Mikroröhre 2.0 mL, Sarstedt AG & Co., Nümbrecht, Germany) and stored at −80 °C until determination of the AA profile.

### 2.5. Amino Acid Analysis

The following AAs were analysed in the feed (pellet and hay) and in the plasma: alanine (Ala), arginine (Arg), asparagine (Asn), aspartic acid (Asp), glutamine (Gln), glutamic acid (Glu), glycine (Gly), histidine (His), isoleucine (Ile), leucine (Leu), lysine (Lys), methionine (Met), phenylalanine (Phe), proline (Pro), serine (Ser), threonine (Thr), tryptophan (Trp), tyrosine (Tyr), valine (Val) and hydroxyproline (Hyp).

The AA composition of the ration components (pellets and hay) was analysed by a GMP+ certified laboratory (NutriControl, Veghel, The Netherlands) and the AA composition of the collagen supplement was provided by SONAC Hydro-P Premium^®^, Darling Ingredients, Son, The Netherlands (Table 2). The AA intake of the horses on blood sampling days for the different dose regimes is calculated in g/day (Table 3).

Amino Acids (AAs) in plasma were extracted by mixing 10 μL of plasma with 1 mL of lysis buffer containing methanol/acetonitrile/dH_2_O (2:2:1). The samples were centrifuged at 16,000× *g* for 15 min at 4 °C to remove cell debris and proteins, and supernatants were collected for liquid chromatography–mass spectrometer (LC–MS) analysis.

LC–MS analysis was performed on an Exactive mass spectrometer (Thermo Scientific^®^, Waltham, MA, USA) coupled to a Dionex Ultimate 3000 autosampler and pump (Thermo Scientific^®^).

AA were separated using a Sequant ZIC-pHILIC column (2.1 × 150 mm, 5 mm, guard column 2.1 × 20 mm, 5 mm; Merck, Darmstadt, Germany) using a linear gradient of acetonitrile (A) and eluent B (20 mM (NH_4_)2CO_3_, 0.1% NH_4_OH in ULC/MS grade water (Biosolve^®^, Valkenswaard, The Netherlands).

Flow rate was set at 150 mL/min and the gradient ran from 20% B to 60% B, followed by a wash step and equilibration at 20% B. The MS operated in polarity-switching mode with spray voltages of 4.5 kV and −3.5 kV. AAs were identified based on exact mass within 5 ppm and further validated by concordance with retention times of standards.

Analysis was performed using Tracefinder software 5.0 (Thermo Scientific^®^). Identification was based on the exact mass within 5 ppm and validated by concordance with retention times of standards. Quantification was based on peak area and AA standard calibration curves.

The samples were measured in three batches. Before, during and after the batches, Quality Control samples were measured to verify signal stability over time.

Hydroxyproline (Hyp) concentrations were analysed only in plasma and at a later stage at the expected peak of t = 2, based on the results of the other AA.

### 2.6. Statistical Analysis

Each amino acid concentration in plasma was analysed using a linear mixed-effects model, with horse and time as random effects and with supplement, time, day and the supplement–time interaction as fixed effects. A normal probability plot on the residuals was used to check for normality. Akaike’s information criterion was used for model reduction. For the effects in the final model, 95% profile (log) likelihood confidence intervals were calculated.

As the study was performed in a randomised crossover design, with a maximal level of standardisation regarding stabling, management and further diet, any possible day interactions did not need to be considered in the statistical analysis.

## 3. Results

The horses remained healthy during the study period. The pellets with the collagen supplement were fully consumed and therefore considered palatable. Comparing the body weight measurements on test day 0 and 22, all horses gained weight (mean 19.5 kg). The CH dose regimen was not adjusted for body weight because all horses had a starting body weight of approximately 600 kg.

All AA plasma concentrations increased after consumption of the feed supplements. Comparing all AA plasma concentrations, horses receiving the dose C_H_ reached the highest maximum plasma concentrations.

Using 95% profile (log) likelihood confidence intervals (CI), there were 6 AA (Ala, Arg, Gln, Gly, Pro, Ser) with statistical differences between the mean plasma concentrations reached with C_L_ and C_H_ compared to the control dose C_N_ over all sampling times (Table 4). For the remaining AAs there were no statistical differences between the mean plasma concentrations.

The data did not show a supplement–time interaction. Therefore, the supplement effects were the same at all timepoints. Horses fed the high supplement dose still showed increased plasma AA concentrations at t0, having received the high dose for the previous 6 days. For all AAs with statistical differences in plasma concentrations between C_H_ and C_N_, the absolute plasma concentrations at t0 were higher for the C_H_- than for C_N_- group. This indicates that 23 h after ingestion of the FS, the AA plasma concentrations (at t0) measured after 100 g of HC are statistically higher than the concentrations measured with the control dose C_N_.

Most peak plasma concentrations were reached at t2. Peak plasma concentrations were reached for 15/19 AA two hours (t2) after feeding dose C_H_, and 13/19 AA also peaked two hours (t2) after feeding dose C_L_. Therefore, hydroxyproline concentrations, which were measured at a later stage, were only determined for t0 and t2.

**Figure 1:**
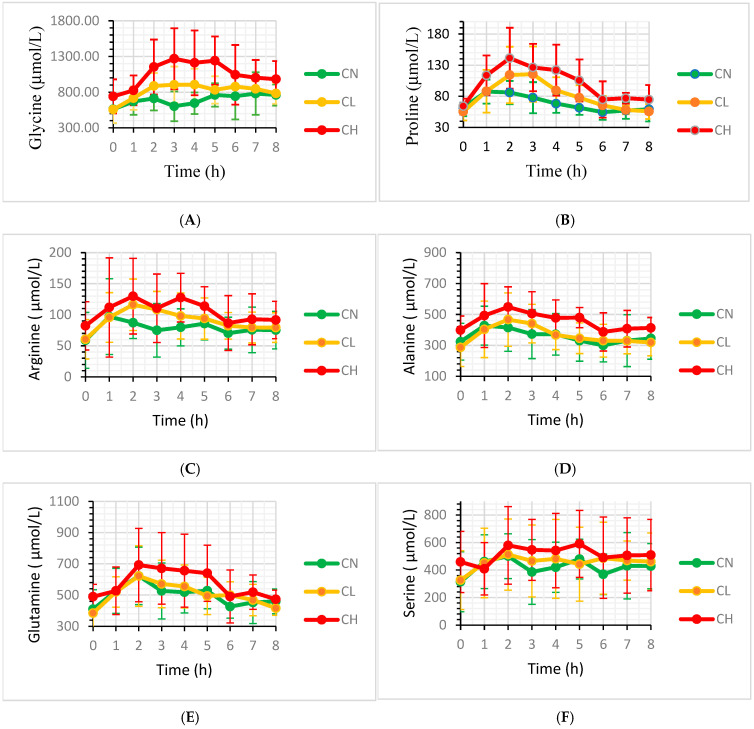
(**A**–**F**) Mean AA plasma concentrations (µmol/L ± s.d.) on day 7, 14 and 21 at t = 0–8 of horses that received no hydrolysed collagen (HC) supplement (C_N_ (0 g/d) or were supplemented with 50 (C_L_) or 100 (C_H_) g/d HC.

The curve of the mean plasma concentration and standard deviation of the six AA with statistical differences between dose C_L_/C_H_ and C_N_ are presented in Figure 1A–F for t0–8(h) on day 7, 14 and 21. Individual variances in AA plasma concentrations can be observed for all horses. For 5 of the 6 AAs (Pro, Arg, Ala, Gln and Ser), the curves show a slight increase in plasma concentrations at t7.

## 4. Discussion

In this study horses received a control or one of two different doses of a dietary supplement which contained hydrolysed collagen. This dietary supplement, rich in alanine, glutamine, glycine, proline and hydroxyproline, resulted in significantly elevated plasma concentrations of glycine, proline, alanine, arginine, glutamine and serine, indicating adequate oral absorption. The plasma concentration of hydroxyproline was measured at the expected peak concentration two hours post consumption and was also shown to be statistically elevated. In previous studies hydroxyproline was not or could not be measured [18,25]. In this study a correlation was shown between the supplement dose and plasma AA concentrations. A dose of 100 g HC once daily resulted in higher plasma concentrations, which also could be detected for at least 24 h.

Data showed that the horses that were fed the high supplement dose still had increased plasma AA concentrations at t0, having received the high dose for the previous 6 days. This means that the horses reached elevated AA concentrations which were maintained for at least 24 h after six days of feeding 100 g HC.

Coenen et al. [25] reported comparable findings, demonstrating that seven days of gelatin supplementation was sufficient to elevate free glycine and proline concentrations in equine blood, remaining nearly unchanged thereafter.

In our study maximal plasma concentrations were reached 120 min after consumption of the FS for 5 of the 6 AAs, with statistical differences between supplement doses. The only exception was Glycine with peak concentrations at t4 for C_L_ and t3 for C_H_. Coenen et al. [25] reported comparable results, with peak concentrations of asparagine, arginine and lysine being measured at 2 h after feeding of the gelatin supplement. Ornithine peaked after four hours and, as in our study, glycine peaked after 3 h.

These peaks point to the short absorption times as result of pre-caecal digestion of AAs in horses [22]. Metayer et al. [30] described a T_50_ for gastric emptying of approximately 145 min after a small high starch meal, showing that the results in the present study are comparable to previous studies [25,30].

For 5 of the 6 AAs with statistical differences between the C_H_ and C_N_ plasma, concentrations slightly increased again 7 h after feeding (Figure 1B–F). This increase might be a result of some delayed gastric emptying or delayed uptake. Feeding no forage during the blood sampling may influence gastric motility and gastric emptying, which in turn could affect the bioavailability of amino acids (AAs) or lead to an increase in tissue-derived AA. A previous study in fasting horses demonstrated a peak in plasma concentrations of several AAs at 5 h post feeding [31]. Alternatively, the collagen uptake itself might lead to dietary interactions that could affect gastric emptying [32].

There is also some evidence to suggest that the large intestine might play a small role in the uptake of AA, as AA transporters are present in the mucosa of the cecum and colon [23]. However, most studies suggest that the majority of AA absorption occurs in the small intestine and that the evidence to support the absorption of AA in the large intestine is minimal [33,34,35].

The amino acid composition of collagen hydrolysates depends on the mechanism of production and the origin of the collagen. Collagen molecules consist in their primary structure of a repeating glycin-X-Y sequence [36]. The X and Y position are often filled by proline and hydroxyproline molecules [37,38].

In the study by Ao and Li [39], the authors described that collagen hydrolysates are mainly composed of Gly, Pro, Glu, Hyp and Ala residues. Hydrolysis of collagen with different proteases also leads to different AA composition of the hydrolysates [39]. This would explain why the concentration of AAs such as Arg can be high in HC supplements, even if this AA is not generally one of the main components of collagen hydrolysates. Coenen et al. [25] showed that the inclusion of 30 g gelatin (a hydrolysed collagen) into a standard test meal resulted in significantly higher post-prandial plasma AA concentrations of Gly and Pro in comparison to the control. Ala and Ser were also increased, but the effect was less marked. The plasma curve of Gln was not reported in Coenen’s study. Coenen et al. [25] also reported significantly higher plasma concentrations of Val and Orn. In the present study, however, no statistical difference for Val was observed and Orn was not included in the analysis.

The limitations of this study include the small study population, limiting the external validity and generalisability. The effects of potential confounders were minimised by a maximal standardisation in stabling, management and diet, randomisation and the cross-over design of the study with the horses serving as their own control.

The large SD in AA plasma concentrations and confidence intervals of the DoM between C_H_/C_N_ and C_L_/C_N_ indicates large individual variation in oral absorption of the supplement in horses.

It is possible that the composition of the hay varied slightly in its AA composition during the study. The AA analysis of the hay was performed on a mixed sample which was collected during the whole study period. In total, the horses received 3 different hay bales from the same pasture, which may have differed in their AA composition. Consequentially, the total daily AA intake might have varied slightly between hay bales. On blood sampling days this inaccuracy was reduced as the horses received their last forage meal 12 h prior to sampling at t0.

In this study it must be further considered that only free AAs were measured, and that the results are product specific. The oral absorption of one specific HC dietary supplement was tested, and it is therefore not possible to draw conclusions about the oral absorption and the resulting AA plasma concentrations of other dietary supplements containing HC, as these might differ in exact amino acid composition.

## 5. Conclusions

This study demonstrated the palatability and availability of bioactive peptides from the hydrolysed collagen of this specific dietary supplement. There was a correlation between supplement dose and plasma AA concentration. Although clinical efficacy was not evaluated in this study, a dose of 100 g HC once daily resulted in higher plasma concentrations, which could be detected for at least 24 h, suggesting greater potential clinical relevance.

## Figures and Tables

**Table 1:** Study design.

Day	Activity	Details
1–6	Dose regime (C_N_, C_L_, C_H_) ^1^	Normal feeding schedule ^3^ IV catheters placed evening day 6
7	Blood sampling day ^2^	Blood sample T = 0 Feeding HC dose regime only (C_N_, C_L_, C_H_) ^1^ Blood samples T = 1–8 h after HC supplement
8–13	Dose regime 2 (C_N_, C_L_, C_H_) ^1^	Normal feeding schedule ^3^ IV catheters placed evening day 13
14	Blood sampling day ^2^	Blood sample T = 0 Feeding HC dose regime only (C_N_, C_L_, C_H_) ^1^ Blood samples T = 1–8 h after HC supplement
15–20	Dose regime 3 (C_N_, C_L_, C_H_) ^1^	Normal feeding schedule ^3^ IV catheters placed evening day 20
21	Blood sampling day ^2^	Blood sample T = 0 Feeding HC dose regime only (C_N_, C_L_, C_H_) ^1^ Blood samples T = 1–8 h after HC supplement

^1^ C_N_ = concentration none (0 g/d hydrolysed collagen), C_L_ = concentration low (50 g/d hydrolysed collagen), C_H_ = concentration high (100 g/d hydrolysed collagen); ^2^ Plasma AA were evaluated on test days 7, 14 and 21 for t = 0–8 (h); ^3^ Normal feeding schedule: 7:00: 3 kg hay, 8:00: HC dose regime, 13:00: 3 kg hay and mineral/vitamin supplement, 20:00: 6 kg hay.

**Table 2:** Amino Acid composition of ration components (pellets ^1^, hay ^1^ and HC supplement ^2,3^).

AA	Pellet I ^1^ g/kg	Pellet II ^1^ g/kg	Hay ^1^ g/kg	HC ^2^ g/16 gN
Ala	5.3	11.9	3.2	8.9
Arg	8.3	13.0	2.6	7.6
Asp	8.7	12.1	4.4	5.8
Cys	2.1	1.9	0.6	0.1
Glu	23.0	28.9	5.3	10.5
Gly	5.7	21.6	2.8	19.9
His	2.6	2.9	0.9	0,8
Ile	4.0	4.6	2.2	1.3
Leu	7.5	9.2	4.0	3.4
Lys	4.6	6.8	2.6	3.5
Met	1.9	2.3	1.5	8.0
Phe	4.8	5.9	2.4	2.3
Pro	7.1	15.8	2.8	10.9
Ser	5.0	6.8	2.1	3.2
Thr	3.9	5.0	2.4	2.0
Tyr	3.4	3.7	1.6	0.9
Val	5.8	7.3	2.9	2.7
Hyp	<0.5	9.8	<0.5	11.2
Trp				0.1

^1^ Analysed by NutriControl, Veghel, The Netherlands; ^2^ SONAC Hydro-P Premium^®^, analysis provided by Darling Ingredients, Son, The Netherlands; ^3^ AA analysis was not performed for the Vitamin and mineral supplement (Subli Mineralenbikkels^®^, Voermeesters B.V. Wageningen, The Netherlands); crude protein content 108 g/kg.

**Table 3:** Amino Acid intake (g/day) on blood sampling days (7, 14, 21) of the horses supplemented with hydrolysed collagen for the dose regimes (C_N_, C_L_, C_H_) ^1^.

AA	C_N_ Dose g/day	C_L_ Dose g/day	C_H_ Dose g/day
Ala	5.3	9.2	13.1
Arg	8.3	11.3	14.3
Asp	8.7	11.0	13.3
Cys	2.1	2.1	2.1
Glu	23	27.4	31.8
Gly	5.7	14.7	23.8
His	2.6	2.9	3.2
Ile	4.0	4.5	5.1
Leu	7.5	8.8	10.1
Lys	4.6	6.0	7.5
Met	1.9	2.2	2.5
Phe	4.8	5.7	6.5
Pro	7.1	12.2	17.4
Ser	5.0	6.2	7.5
Thr	3.9	4.7	5.5
Tyr	3.4	3.7	4.1
Val	5.8	6.9	8.0
Hyp	<0.5	5.4	10.8

^1^ C_N_ = concentration none (0 g/d hydrolysed collagen), C_L_ = concentration low (50 g/d hydrolysed collagen), C_H_ = concentration high (100 g/d hydrolysed collagen)

**Table 4:** Statistically relevant differences of AAs in μmol/L compared to C_N_^1^, presenting DoM (difference of means) with a 95% (log) likelihood confidence interval (CI) ^2^.

AA	Dose C_H_^1^	Dose C_L_^1^
Ala	99.27 (76.00–122.54)	-
Arg	26.86 (16.32–37.40)	12.08 (1.54–22.62)
Gln	76.24 (39.86–112.62)	-
Gly	359.10 (288.86–429.33)	118.97 (48.74–189.21)
Pro	0.35 (0.27–0.43)	0.12 (0.04–0.21)
Ser	93.49 (51.19–135.79)	-
Hyp ^3^	59.66 (49.73–69.59)	36.68 (26.75–46.61)

^1^ C_N_ = 0 g/d hydrolysed collagen, C_L_ = 50 g/d hydrolysed collagen, C_H_ = 100 g/d hydrolysed collagen.; ^2^ Confidence interval excluding 0 is considered statistically relevant; ^3^ hydroxyproline was analysed only at t = 2.

## Data Availability

The raw data supporting the conclusions of this article will be made available by the authors on request.

## Supplementary Materials

The following supporting information can be downloaded at https://www.mdpi.com/article/10.3390/ani15213195/s1: Table S1: Total daily intake of energy and protein composition per dosage regime per kg bwt. Table S2: Energy content and protein composition of the feeding components per kg product.

## Abbreviations

The following abbreviations are used in this manuscript:

AA	Amino Acids
ALA	Alanine
ARG	Arginine
ASN	Asparagine
ASP	Aspartic acid
bwt	Body weight
C_H_	Concentration high hydrolysed collagen
C_L_	Concentration low hydrolysed collagen
C_N_	Concentration none hydrolysed collagen (control)
d	Day
DoM	Differences of means
EGUS	Equine Gastric Ulcer Syndrome
EW-pa	Energy value horse (Net Energy)
FS	Feeding supplement
GLN	Glutamine
GLU	Glutamic acid
GLY	Glycine
HC	Hydrolysed collagen
HIS	Histidine
HYP	Hydroxyproline
ILE	Isoleucine
kg	Kilogram
LEU	Leucine
LYS	Lysine
MET	Methionine
PHE	Phenylalanine
PRO	Proline
SER	Serine
THR	Threonine
TRP	Tryptophan
TYR	Tyrosine
VAL	Valine

## References

[1] Bello A.E., Oesser S. (2006). Collagen Hydrolysate for the Treatment of Osteoarthritis and Other Joint Disorders:A Review of the Literature. Curr. Med. Res. Opin..

[2] Benito-Ruiz P., Camacho-Zambrano M.M., Carrillo-Arcentales J.N., Mestanza-Peralta M.A., Vallejo-Flores C.A., Vargas-López S.V., Villacís-Tamayo R.A., Zurita-Gavilanes L.A. (2009). A Randomized Controlled Trial on the Efficacy and Safety of a Food Ingredient, Collagen Hydrolysate, for Improving Joint Comfort. Int. J. Food Sci. Nutr..

[3] Crowley D.C., Lau F.C., Sharma P., Evans M., Guthrie N., Bagchi M., Bagchi D., Dey D.K., Raychaudhuri S.P. (2009). Safety and Efficacy of Undenatured Type II Collagen in the Treatment of Osteoarthritis of the Knee: A Clinical Trial. Int. J. Med. Sci..

[4] Kumar S., Sugihara F., Suzuki K., Inoue N., Venkateswarathirukumara S. (2015). A Double-Blind, Placebo-Controlled, Randomised, Clinical Study on the Effectiveness of Collagen Peptide on Osteoarthritis. J. Sci. Food Agric..

[5] Lugo J.P., Saiyed Z.M., Lane N.E. (2016). Efficacy and Tolerability of an Undenatured Type II Collagen Supplement in Modulating Knee Osteoarthritis Symptoms: A Multicenter Randomized, Double-Blind, Placebo-Controlled Study. Nutr. J..

[6] Luo C., Su W., Song Y., Srivastava S. (2022). Efficacy and Safety of Native Type II Collagen in Modulating Knee Osteoarthritis Symptoms: A Randomised, Double-Blind, Placebo-Controlled Trial. J. Exp. Orthop..

[7] Moskowitz R.W. (2000). Role of Collagen Hydrolysate in Bone and Joint Disease. Semin. Arthritis Rheum..

[8] Clark K.L., Sebastianelli W., Flechsenhar K.R., Aukermann D.F., Meza F., Millard R.L., Deitch J.R., Sherbondy P.S., Albert A. (2008). 24-Week Study on the Use of Collagen Hydrolysate as a Dietary Supplement in Athletes with Activity-Related Joint Pain. Curr. Med. Res. Opin..

[9] Beynen A.C., Geene H.W.V., Grim H.V., Jacobs P., Vlerk T.V. (2010). der Oral Administration of Gelatin Hydrolysate Reduces Clinical Signs of Canine Osteoarthritis in a Double-Blind, Placebo-Controlled Trial. Am. J. Anim. Vet. Sci..

[10] Blees N.R., Teunissen M., Dobenecker B., Prawitt J., Tryfonidou M.A., Jan Corbee R. (2025). Collagen Hydrolysates as Nutritional Support in Canine Osteoarthritis: A Narrative Review. J. Anim. Physiol. Anim. Nutr..

[11] Comblain F., Barthélémy N., Lefèbvre M., Schwartz C., Lesponne I., Serisier S., Feugier A., Balligand M., Henrotin Y. (2017). A Randomized, Double-Blind, Prospective, Placebo-Controlled Study of the Efficacy of a Diet Supplemented with Curcuminoids Extract, Hydrolyzed Collagen and Green Tea Extract in Owner’s Dogs with Osteoarthritis. BMC Vet. Res..

[12] Gupta R.C., Canerdy T.D., Lindley J., Konemann M., Minniear J., Carroll B.A., Hendrick C., Goad J.T., Rohde K., Doss R. (2012). Comparative Therapeutic Efficacy and Safety of type-II Collagen (uc-II), Glucosamine and Chondroitin in Arthritic Dogs: Pain Evaluation by Ground Force Plate. J. Anim. Physiol. Anim. Nutr..

[13] Gupta R.C., Canerdy T.D., Skaggs P., Stocker A., Zyrkowski G., Burke R., Wegford K., Goad J.T., Rohde K., Barnett D. (2009). Therapeutic Efficacy of Undenatured Type-II Collagen (UC-II) in Comparison to Glucosamine and Chondroitin in Arthritic Horses. J. Vet. Pharmacol. Ther..

[14] Van De Water E., Oosterlinck M., Dumoulin M., Korthagen N.M., Van Weeren P.R., Van Den Broek J., Everts H., Pille F., Van Doorn D.A. (2017). The Preventive Effects of Two Nutraceuticals on Experimentally Induced Acute Synovitis. Equine Vet. J..

[15] Butler K.D., Hintz H.F. (1977). Effect of Level of Feed Intake and Gelatin Supplementation on Growth and Quality of Hoofs of Ponies. J. Anim. Sci..

[16] Dobenecker B., Reese S., Jahn W., Schunck M., Hugenberg J., Louton H., Oesser S. (2018). Specific Bioactive Collagen Peptides (PETAGILE^®^) as Supplement for Horses with Osteoarthritis: A Two-Centred Study. J. Anim. Physiol. Anim. Nutr..

[17] Andrews F., Camacho-Luna P., Lamp B., Olijve J. (2017). Effects of Hydrolyzed Collagen on Equine Gastric Ulcers Scores and Gastric Juice pH. J. Equine Vet. Sci..

[18] Camacho-Luna P., Andrews F.M., Keowen M.L., Garza Jr F., Liu C.-C., Lamp B., Olijve J. (2022). The Effect of Porcine Hydrolysed Collagen on Gastric Ulcer Scores, Gastric Juice pH, Gastrin and Amino Acid Concentrations in Horses. Equine Vet. Educ..

[19] Castro G.A., Carvalho J.E., Tinti S.V., Possenti A., Sgarbieri V.C. (2010). Anti-Ulcerogenic Effect of a Whey Protein Isolate and Collagen Hydrolysates against Ethanol Ulcerative Lesions on Oral Administration to Rats. J. Med. Food.

[20] Kumar D., Hegde H.V., Patil P.A., Roy S., Kholkute S.D. (2013). Antiulcer Activity of Water Soaked *Glycine max* L. Grains in Aspirin Induced Model of Gastric Ulcer in Wistar Rats. J. Ayurveda Integr. Med..

[21] Bakaeva Z.V., Sangadzhieva A.D., Tani S., Myasoedov N.F., Andreeva L.A., Torshin V.I., Wallace J.L., Tanaka T. (2016). Glyprolines Exert Protective and Repair-Promoting Effects in the Rat Stomach: Potential Role of the Cytokine GRO/CINC-1. J. Physiol. Pharmacol..

[22] Mok C.H., Urschel K.L. (2020). Amino Acid Requirements in Horses. Asian-Australas. J. Anim. Sci..

[23] Woodward A.D., Holcombe S.J., Steibel J.P., Staniar W.B., Colvin C., Trottier N.L. (2010). Cationic and Neutral Amino Acid Transporter Transcript Abundances Are Differentially Expressed in the Equine Intestinal Tract. J. Anim. Sci..

[24] Iwai K., Hasegawa T., Taguchi Y., Morimatsu F., Sato K., Nakamura Y., Higashi A., Kido Y., Nakabo Y., Ohtsuki K. (2005). Identification of Food-Derived Collagen Peptides in Human Blood after Oral Ingestion of Gelatin Hydrolysates. J. Agric. Food Chem..

[25] Coenen M., Appelt K., Niemeyer A., Vervuert I. (2006). Study of Gelatin Supplemented Diet on Amino Acid Homeostasis in the Horse. Equine Vet. J..

[26] Henneke D.R., Potter G.D., Kreider J.L., Yeates B.F. (1983). Relationship between Condition Score, Physical Measurements and Body Fat Percentage in Mares. Equine Vet. J..

[27] Sykes B.W., Hewetson M., Hepburn R.J., Luthersson N., Tamzali Y. (2015). European College of Equine Internal Medicine Consensus Statement—Equine Gastric Ulcer Syndrome in Adult Horses. J. Vet. Intern. Med..

[28] Centraal Veevoederbureau (2004). Het EW-Pa en VREp Systeem.

[29] Harris P.A., Ellis A.D., Fradinho M.J., Jansson A., Julliand V., Luthersson N., Santos A.S., Vervuert I. (2017). Review: Feeding Conserved Forage to Horses: Recent Advances and Recommendations. Animal.

[30] Métayer N., Lhôte M., Bahr A., Cohen N.D., Kim I., Roussel A.J., Julliand V. (2004). Meal Size and Starch Content Affect Gastric Emptying in Horses. Equine Vet. J..

[31] Russell M.A., Rodiek A.V., Lawrence L.M. (1986). Effect of Meal Schedules and Fasting on Selected Plasma Free Amino Acids in Horses. J. Anim. Sci..

[32] Grasset E., Briand F., Virgilio N., Schön C., Wilhelm M., Cudennec B., Ravallec R., Aboubacar H., Vleminckx S., Prawitt J. (2024). A Specific Collagen Hydrolysate Improves Postprandial Glucose Tolerance in Normoglycemic and Prediabetic Mice and in a First Proof of Concept Study in Healthy, Normoglycemic and Prediabetic Humans. Food Sci. Nutr..

[33] Urschel K.L., Lawrence L.M. (2013). Amino Acids and Protein. Equine Applied and Clinical Nutrition.

[34] Bochröder B., Schubert R., Bödeker D. (1994). Studies on the Transport in Vitro of Lysine, Histidine, Arginine and Ammonia across the Mucosa of the Equine Colon. Equine Vet. J..

[35] Bockisch F., Taubert J., Coenen M., Vervuert I. (2023). Protein Evaluation of Feedstuffs for Horses. Animals.

[36] Hulmes D.J.S., Fratzl P. (2008). Collagen Diversity, Synthesis and Assembly. Collagen.

[37] Gómez-Guillén M.C., Giménez B., López-Caballero M.E., Montero M.P. (2011). Functional and Bioactive Properties of Collagen and Gelatin from Alternative Sources: A Review. Food Hydrocoll..

[38] Schroeder W.A., Kay L.M., LeGette J., Honnen L., Green F.C. (1954). The Constitution of Gelatin. Separation and Estimation of Peptides in Partial Hydrolysates. J. Am. Chem. Soc..

[39] Ao J., Li B. (2012). Amino Acid Composition and Antioxidant Activities of Hydrolysates and Peptide Fractions from Porcine Collagen. Food Sci. Technol. Int..

