# Supplementary material for: Free Plasma Amino Acid Concentrations in Horses Fed Different Dosing Regimens of Hydrolysed Collagen
Source: Animals (Basel). 2025 Nov 3;15(21):3195. doi: 10.3390/ani15213195 (PMC12609581; doi:10.3390/ani15213195)
Supplement: Supplementary file 1 [file animals-15-03195-s001.zip › animals-3897423-supplementary.pdf]

Table S1: Total daily intake of energy and protein composition per dosage regime (C_N, C_L, C_H)[1] per kg bwt[2] on day 1-6, 8-13 and 15-20.

Nutrient	C_N	C_L	C_H
EW-pa3 (/kg)	0.011	0.011	0.011
Digestible crude protein (g/kg)	1.170	1.171	1.171
Crude protein (g/kg)	4.110	4.163	4.248

[1] C_N = concentration none (0 g/d hydrolysed collagen), C_L= concentration low (50 g/d hydrolysed collagen), C_H = concentration high (100 g/d hydrolysed collagen).

[2] btw= body weight in kg. 3 EW-pa = energy value horses per kg product

Table S2: Energy content and protein composition of the feeding components (roughage, pellet feed I, pellet feed II, mineral/vitamin supplement) per kg product.

Nutrient	Hay	Pellet feed I	Pellet feed II	M/Vsupplement[1]
EW-pa[2] (/kg)	00.45	00.80	00.72	00.74
Digestible crude protein (g/kg)	48.00	80.00	73.00	77.00
Crude protein (g/kg)	65.68	114.00	197.00	108.00

[1] M/V supplement= mineral/vitamin supplement.

[2] EW-pa= energy value horse per kg product
